# Investigating Correlates of Self-Regulation in Early Childhood with a Representative Sample of English-Speaking American Families

**DOI:** 10.1007/s10826-012-9595-z

**Published:** 2012-05-13

**Authors:** Jessica Taylor Piotrowski, Matthew A. Lapierre, Deborah L. Linebarger

**Affiliations:** 1 Department of Communication Science, The Amsterdam School of Communication Research ASCoR, University of Amsterdam, Kloveniersburgwal 48, 1012 CX Amsterdam, The Netherlands; 2 Annenberg School for Communication, University of Pennsylvania, Philadelphia, PA USA; 3 Department of Teaching and Learning, College of Education, University of Iowa, Iowa City, IA USA

**Keywords:** Demographic groups, Early childhood, Parenting style, Representative, Self-regulation

## Abstract

Children who possess less self-regulatory skill are at a disadvantage when compared to children who demonstrate greater skill at regulating their emotions, cognitions and behavior. Children with these regulatory deficits have difficulty connecting with peers, generating relationships with teachers, negotiating their social world, and succeeding academically. By understanding the correlates of self-regulatory abilities, interventions can be developed to ensure that children at-risk for poor self-regulation receive the support necessary to enhance their regulatory skills. Using data from a nationally representative survey of English-speaking American parents with children between the ages of two and eight (*n* = 1,141), we evaluated a host of demographic and parenting variables to isolate the correlates of self-regulation. Older children were found to have fewer regulatory problems than younger children while children from low-income homes and male children were found to have greater problems with self-regulation. Minority status, household composition (single vs multi-parent), and parental education were not significant correlates of self-regulation. Findings also illustrate the powerful relationship between parenting style and self-regulation. Parents who rely on nurturing parenting practices that reinforce the child’s sense of autonomy while still maintaining a consistent parenting presence (i.e., authoritative parenting) have children who demonstrate stronger self-regulatory skills. Parents who exert an excess of parental control (i.e., authoritarian parents) have children with weaker self-regulatory skills. And lastly, parents who have notable absence of control (i.e., permissive parents) are more likely to have children with considerable regulatory deficits. Results offer implications for both practitioners and scholars.

## Introduction

Over the past three decades, our understanding of children’s social and cognitive development has experienced dramatic growth. This body of research has highlighted the interconnected roles that child, family, school, and larger sociocultural factors play on development (Bronfenbrenner and Morris 1998; Morrison et al. 2005). One factor that has emerged as an important predictor of children’s healthy development is self-regulation (Blair 2002; Buckner et al. 2009; McClelland et al. 2007). Defined as a set of acquired, intentional skills involved in controlling, directing, and planning one’s cognition, emotions, and behaviors (Schunk and Zimmerman 1997), successful self-regulation is thought to develop from an interaction between biological factors (e.g., temperament) and experiential factors (e.g., early experiences and social interactions) and is argued to be a marker of adaptive development (Morrison et al. 2009).

Considering the fact that self-regulation is viewed as a critical building block for healthy development, it is no surprise that there is a growing body of literature investigating the predictors of self-regulation. By understanding which characteristics are most frequently and most strongly associated with self-regulatory abilities, interventions can be developed to ensure that children at-risk for poor self-regulation receive the support necessary to enhance their regulatory skills. Recent studies have examined a host of biological and experiential factors to determine whether and how they are linked to self-regulation (see Morrison et al. 2009). Frequently included in these investigations are demographic characteristics of the child, parent, or household (e.g., Evans and Rosenbaum 2008; Matthews et al. 2009) as well as parenting factors (e.g., Crossley and Buckner 2011). These demographic and parenting factors correlate with and/or predict self-regulation. Despite this growing body of research that includes several large-sample studies (e.g., NICHD 2003), there is currently no published research utilizing a nationally-representative probability sample of the American population to investigate correlates of self-regulation in young children. We seek to address this gap by using data from a nationally representative survey of English-speaking American parents whose children were between the ages of 8 months and 8 years. The primary benefit associated with using a randomly chosen representative sample is that it provides researchers the opportunity to generalize their findings to the population it represents (Lohr 2010). In this case, it allows us to detail how American children and their parents behave without having to offer many of the normally mentioned caveats regarding selection bias or response bias (Lohr 2010).

Fielded in the spring 2009, our survey addressed a number of topics including family demographics, parenting style, and children’s regulatory skills. The analyses presented here utilized data from 1,141 parents whose children were between the ages of 2 and 7 years (any child who had not yet turned eight was considered eligible) at the time of the survey. We investigate whether select demographic and parenting variables previously associated with self-regulation remain significant correlates of children’s self-regulation. By looking at the role of these variables in concert with one another, we are able to see which variables have the strongest relationships with self-regulation. Such an understanding can offer important directions for future intervention work designed to support the regulatory skills of young children.

### Factors Predicting Self-Regulation Among Children

Demographic factors (both child and family variables) and parenting behaviors are key predictors of self-regulation. We briefly detail the relevant findings from each category. Then, based on this review of the literature, we posit the relationship we expect to find in our analyses and ask whether this relationship will remain when controlling for the other demographic and parenting variables in our model.

Utilizing a variety of measurement techniques to assess self-regulation, the consensus from numerous studies is that self-regulatory processes grow stronger with age (e.g., Kopp 1982; Raffaelli et al. 2005; Simonds et al. 2007). These skills emerge early in life and increase in sophistication over time. Many researchers believe that the link between age and self-regulation is due to the maturation of the prefrontal cortex (Bunge and Zelazo 2006; Zelazo and Cunningham 2007) and other brain regions (Diamond 2000; Lewis and Todd 2007) as these areas are primarily responsible for controlling cognition and emotion.

Evidence also indicates that significant regulatory differences exist between the genders (Duckworth and Seligman 2006; Kochanska et al. 2001; Matthews et al. 2009; Ponitz et al. 2008). Young boys experience more difficulty controlling their cognitions and behaviors when compared with young girls. These findings extend across methodologies as differences have been found in both objective measurements and teacher report (Matthews et al. 2009). Recently, researchers have posited that the relationship between gender and self-regulation may be moderated by family income. Entwisle et al. (2007) found that boys living in low income families performed significantly worse on tests of reading skills than females living in low income families. These gender differences were not found for children from middle income families. Entwisle et al. (2007) explain that, in low income families, boys are expected to be more interested in rough play and exhibit more extroverted behavior around others while in middle-to-upper income families these expectations do not exist. It is possible that these endorsed and supported stereotypes held by both parents and teachers may contribute to low income boys’ poorly regulated behaviors (Morrison et al. 2009). More research is needed to determine if income does moderate the relationship between gender and self-regulation.

Research on the relationship between child’s race/ethnicity and self-regulatory behaviors remains limited. The available literature indicates that minority status may be a risk factor for lower self-regulation. When controlling for a host of risk variables, Sektnan et al. (2010) found that being African American was significantly correlated with lower self-regulation in kindergarten. Although these researchers did not find any relationship between Hispanic ethnicity and self-regulation, other researchers have found that children from disadvantaged Hispanic families entered preschool with significantly lower regulatory skills than their peers (Wanless et al. 2007 as cited in Sektnan et al. 2010).

Beyond child level demographic variables, evidence also indicates that family level demographic variables are associated with self-regulation. Children whose parents, particularly mothers, have greater formal education are better able to exhibit self-control in social and school settings. Mothers with lower educational attainment have children who perform worse on tests of self-regulation (Evans and Rosenbaum 2008; Sektnan et al. 2010) while other studies have found that higher educational status for *both* parents positively predicted behavioral regulation (McClelland et al. 2007). Children from homes with fewer economic resources are also routinely found to have lower self-regulation skills than their peers from more affluent backgrounds. Evans and Rosenbaum (2008) found that white children living in low-income households in rural communities had lower self-regulation when compared to their higher income peers. In a longitudinal study with a sample of ethnically diverse children, they found similar effects of income status on self-regulation with additional evidence suggesting that the effect of income on regulation was independent of other important secondary variables (e.g., maternal education, ethnicity, single-parent status, Evans and Rosenbaum 2008). Howse et al. (2003) found that children from economically disadvantaged backgrounds had lower self-regulation than their more advantaged peers. Sektnan et al. (2010) similarly found that children with a lower income-to-needs ratio from 1 through 54 months of age had poorer behavioral regulation skills at 54 months even when controlling for other risk variables. They argue that children experiencing risk factors, including socioeconomic hardship, have fewer resources available to promote behavioral regulation and fewer opportunities to practice these skills. Lastly, new research on neural activity also supports the observed relationships between socioeconomic hardship and regulatory behaviors. Researchers have found that financial hardship is associated with alterations in prefrontal cortex functioning and the cognitive processes underlying regulation in children (Kishiyama et al. 2009).

Unlike family income and self-regulation, there is limited empirical research on the relationship between household composition (single vs multi-parent household) and self-regulation. Work by Colman et al. (2006) found that household composition did not significantly predict self-regulation in middle childhood. Other research (Evans and Rosenbaum 2008) suggests that children from multi-parent homes may have stronger regulatory skills than children from single-parent homes, although this relationship disappeared when controlling for socioeconomic status, suggesting that it is not the number of parents in the household that is important but rather it is the economic stress facing single parents that impacts children’s self-regulation (see also Murry and Brody 1999).

Parent practices and styles have also been shown to influence children’s self-regulation skills, although the evidence on what these relationships looks like is mixed. Several studies have found a positive impact of parental controlling behavior on children’s self-regulation skills (Eiden et al. 2001; Feldman and Klein 2003) while others have found that the children of less controlling parents enjoy more success (Kochanska and Knaack 2003; Stansbury and Zimmerman 1999) particularly when parents work to support their child’s sense of autonomy (Bernier et al. 2010). Here, we investigate the relationship between three parenting styles—authoritative, authoritarian, and permissive (Baumrind 1971)—and children’s self-regulation.

Authoritative parenting is characterized as parenting that focuses on teaching, encouraging exploration, and guiding the child’s behavior (Baumrind 1971). Parents who employ an authoritative parenting style help the child work through stressful situations by talking with the child about their frustrations and encouraging them to solve the problem on their own. Grolnick and Ryan (1989) found that mother’s authoritative parenting style was positively related to a child’s self-reported self-regulation. Similarly, research by Tudge et al. (2003) and Crossley and Buckner (2011) positively linked authoritative parenting style with stronger behavioral regulation. A meta-analysis of 41 studies measuring parenting styles and the self-regulation skills of preschoolers revealed a small but consistent positive effect for authoritative parenting (defined as positive control) on gaining children’s compliance (one domain of self-regulation; Karreman et al. 2006).

Parents who exhibit an authoritarian style of parenting frequently use methods that are more restrictive and controlling of the child’s behavior, sometimes with harsh punishment (Karreman et al. 2006). This style of parenting is hypothesized to constrain the development of self-regulation in children, as it interferes with the child’s own ability to sort through complicated emotional and behavioral situations. In the meta-analysis mentioned above, the researchers found a moderate negative effect for authoritarian parenting style (defined as negative control) on children’s compliance (one domain of self-regulation; Karreman et al. 2006). Crossley and Buckner (2011) found a similar pattern. Children from low-income families whose parents utilized harsh parenting practices demonstrated weaker regulatory skills.

As opposed to the previous parenting styles discussed above, permissive parenting is marked by the absence of parental control. Parents classified as permissive will avoid punishing their child, will allow certain transgressions to pass, and do not confront their children regarding their behavior (Baumrind 1971). Baumrind (1967) theorized that these children would have poor impulse control; however, the research on permissive parenting and how it affects self-regulation is virtually non-existent. The lone available study found that parents who were more permissive had children who were more likely to have stronger self-regulation skills (Morris 2003).

### Hypotheses and Research Questions

We expect that as child age and parent education increase, children’s self-regulation skills will also be stronger. We expect that being male, a minority, living in a single-parent household or living in a low income home will be associated with poorer self-regulation. Additionally, based on previous research (Entwisle et al. 2007), we ask whether family income differentially impacts the relationship between gender and self-regulation such that male children from low-income families demonstrate greater self-regulatory difficulties.

Research on parenting and children’s self-regulation suggests that parents must undertake a delicate balancing act with their exercise of parental control (Bernier et al. 2010). At the very start of a child’s life, the parent acts as the primary regulator of behavior. As the infant/toddler moves into early childhood, the burden for regulating behavior, emotions, and cognitions falls onto the child. Parents who focus on guiding their child’s budding abilities to regulate themselves rather than stringently dictating or, at the opposite end, leaving the child to manage on their own, have children who are better at self-regulating (Bernier et al. 2010). With this in mind, we expect that parents who exert either an excess of parental control (i.e., authoritarian parents) or lack of parental control (i.e., permissive parents) will have children who demonstrate weaker self-regulation skills. On the other hand, parents who rely on parenting practices that reinforce the child’s sense of autonomy, yet still maintain a consistent parenting presence (i.e., authoritative parents) will have children who demonstrate stronger self-regulatory skills.

In addition to the anticipated directional relationships described above, we seek to determine which independent variables remain significant correlates of self-regulation when all main effects are entered in the model.

## Methods

### Participants

After receiving approval from the sponsoring institution’s Institutional Review Board, a private survey research firm specializing in telephone surveys administered the survey. The study collected a representative sample of 1,454 American households containing at least one person age 18 and older who was the primary caregiver for a child between the ages of 8 months and 7 years (any child who had not yet turned eight was considered eligible). All interviews were conducted in English. As self-regulation data was only available for children two and older, children younger than two were not included in the analysis (*n* = 313). A total of 1,141 cases were used for these analyses (see Table 1 for sample breakdown).

**Table 1:** Individual and family characteristics of the sample (*n* = 1,141)

Characteristic	Percent in category or mean (95 % CI)
Child age (months)	59.09 (57.04, 61.14)
Child gender = male	52.4 %
Child race	
White—not Latino	58.2 %
African American—not Latino	14.1 %
African American—Latino	0.7 %
White—Latino	15.6 %
Asian	2.8 %
Native American	1.3 %
Other	7.2 %
Parent education	14.22 (13.95, 14.48)
Family income = low income	28.4 %
Household composition = single parent household	16.9 %
Parenting styles	
Authoritative score	4.65 (4.61, 4.69)
Authoritarian score	1.64 (1.59, 1.69)
Permissive score	2.05 (1.98, 2.12)
Survey respondent	
Mother	73.3 %
Father	23.3 %
Other	3.5 %
Home language = English only	91.9 %
Special needs = yes	11.9 %
Childcare = yes	44.2 %
Self-regulation	29.96 (29.09, 30.84)

Self-regulation is coded such that *lower* scores indicate better self-regulation

### Design

A rolling cross-sectional survey using a disproportionate stratified random digit dialing procedure was used to collect a representative sample of English-speaking American households. Administration occurred between January 2009 and March 2009 by trained interviewers. Interviews were stratified to increase the incidence of households with children younger than eight as well as to provide oversamples of low income households and those where the primary caregiver was American Indian. In households where the adult was the primary caregiver for more than one child between 8 months and 7 years of age, the target child was selected by randomly asking the respondent to answer questions about either the child with the most recent or the next birthday. The alternating sampling design was implemented in order to ensure that children between the ages of 8 months and just under 1 year would not be selected disproportionately using only the next birthday method, since these children would be more likely than others to have a birthday in the months immediately following the interview.

Interviewers were provided instructions to maximize response rate and to ensure accurate data collection. They were instructed to encourage participation by emphasizing the social importance of the project and to reassure respondents that their information would be kept confidential. In order to maximize survey response, the survey firm enacted several procedures including (1) instituting a call rule of original plus up to 16 callbacks, (2) varying the times of day and days of week in which call-backs were placed, (3) explaining the purpose of survey and expected length, and by (4) allowing respondents to schedule the call-back. The response rate was similar to other nationally representative surveys that have targeted parents of young children 39.1 % (e.g., 40 % for Rideout et al. 2003).

Survey data were weighted to adjust for the fact that not all survey respondents were selected with the same probability and to account for gaps in coverage and non-response biases in the survey frame. Design weights were used to compensate for the known biases from telephone interviewing in general and the unique sample design of the survey, specifically. The resulting design weights were post-stratified along several dimensions obtained from the 2009 national estimates of the Census’ American Community Survey.

### Procedure

After eligibility screening was completed and informed consent received, parents were asked a series of questions including an assessment of the child’s self-regulation skills, parenting behaviors, and household demographics. On average, participants required 50 min to complete the survey. Participants who completed the survey using a landline (about 96 %) were compensated $25.00 while participants completing the survey using a cell phone were compensated $50.00. Cell phone participants were compensated a greater amount in order to offset the costs associated with using their mobile phone minutes. At the conclusion of the survey, participants were provided with contact information for the study coordinator as well as for the Institutional Review Board.

### Measures

The first seven items below measure the hypothesized independent variables (i.e., child’s age, child’s gender, child’s race/ethnicity, parent education, family income, household composition, parenting style) in the models. The next four items are covariates in the models (i.e., survey respondent, home language, special needs, childcare). Finally, the last item measures the dependent variable in all models (i.e., self-regulation).

#### Child Age

Survey participants were asked to indicate the birth date of the target child in the study. Based upon when the data was collected, the child’s age in months was calculated, *M* = 59.09, SD = 35.33.

#### Child Gender

Respondents were asked to report the gender of the participating child. Fifty-two percent of the target children were male. For all analyses, females served as the reference category.

#### Child Race/Ethnicity

Caregivers were asked to report the race of the target child. Rather than using a mutually exclusive measure of race, parents indicated whether the child was a member of a specific racial group and were free to indicate that the child belonged to multiple racial groups. Similar to the way that the United States census assesses Latino background, respondents were also asked to separately indicate whether their child ethnically identified as Latino or Hispanic. Responses to these two questions were then used to create a mutually exclusive race/ethnicity variable. For example, parents who reported that their child was African-American but did not belong to another racial group and were not considered Latino, were classified as African American/non-Latino. Fifty-eight percent of parents in our sub-sample indicated that their child was White/non-Latino, 16 % indicated that their child was White/Latino, 1 % indicated that the child was African American/Latino, 14 % stated that their child was African American/non-Latino, 3 % stated that their child was Asian, 1 % indicated that their child was of Native American decent and 7 % reported that their child was of either multiple racial backgrounds, of a another racial background (e.g., Middle Eastern, Hawaiian) or refused to reply. For all analyses, the White/non-Latino group served as the reference category.

#### Parent Education

Survey respondents were asked how much formal education they, as well as other adult caregivers living in the home, had. The potential answers ranged from 1 (didn’t go to school) to 10 (Ph.D, M.D., J.D., etc.). In order to make the parent’s reported education more interpretable, the values were recoded to approximate how many formal years of schooling each parent had. For example, parents with no formal education were assigned a score of zero, parents with a high school degree/GED were assigned a score of 12 while parents with a master’s degree were assigned a score of 18. Responses were averaged to create an indicator of parent education. The average household in our sample had the equivalent of an associate’s degree, *M* = 14.22, SD = 4.48.

#### Family Income

An income-to-needs ratio, a per capita index adjusted annually for costs of living that reflects absolute income as a proportion of the federal poverty line, was calculated for each participating family. In order to calculate the family’s income-to-needs ratio, participants were asked to report their yearly income as well as the number of adults and children living in the home. This data was used in conjunction with the 2008 poverty threshold data provided by the US Census Bureau. To facilitate comparisons with previous research, the data were dichotomized to reflect low-income versus not low-income. Families under 185 % of the federal poverty level were classified as low income (28 %). For all analyses, non-low-income families served as the reference category.

#### Household Composition

Participants were asked how many people over the age of 18 were living in the house and acted as a parent to the target child. Respondents who indicated that there was only one adult in the home who acted as a caregiver were classified as single parent homes (17 %) while the remainder was classified as multi-parent homes. For all analyses, multi-parent homes served as the reference category.

#### Parenting Styles

Based on Baumrind’s conceptualization of parenting styles (Baumrind 1971), a parent style questionnaire was administered to all participants. The original questionnaire, developed by Robinson et al. (1995), consisted of 62 items. Due to time constraints, three subscales of this measure were administered. The first subscale, warmth and involvement, measured authoritative parenting via 7 items (e.g., when your child is hurt how often do you show sympathy; α = 0.83). The second subscale, non-reasoning punitive strategies, measured authoritarian parenting via 6 items (e.g., how often do you punish without explaining the offense; α = 0.70). The third subscale, follow through, measured permissive parenting via 7 items (e.g., how often does parent find it difficult to discipline the child: α = 0.73). All items were measured on a 5-point Likert scale 1 (never) to 5 (always). For each subscale, a mean across items was calculated (authoritative: *M* = 4.65, SD = 0.69; authoritarian: *M* = 1.64, SD = 0.86; permissive: *M* = 2.05, SD = 1.21). Due to deviations from normality, we used a reflective inverse transformation for the authoritative parenting scale and dichotomized the authoritarian parenting scale (1 = greater authoritarian parenting, divided at median; Tabachnick and Fidell 2007).

#### Survey Respondent

All survey respondents were asked about their relation to the target child. Of those who did not indicate that they were the child’s mother, 23 % reported that they were the father or were a father figure. The remainder (3.5 %) stated that they were related to the child in another way. We created two variables based on responses to this question. One variable indicated that the respondent was the father (coded as 1, other as 0), while the other indicated that the respondent was not the mother or father (coded as 1, other as 0).

#### Home Language

While all interviews took place in English, participants were asked what language was spoken most in the home. Respondents who said English were assigned a value of 1 (92 %) while all others received a value of 0.

#### Special Needs

Respondents were asked whether the target child in the study had been identified with any disabilities or had special learning needs. Respondents who reported that their child had an identified disability were assigned a value of 1 (12 %). Disabilities included visual, hearing, or language impairments; traumatic brain injury; health impairments; and developmental delays.

#### Childcare

Participants were asked whether the target child was enrolled in a childcare setting or attended after school care. Respondents who said that that their child was enrolled in one of these settings were assigned a value of 1 (44 %) while others were assigned a value of 0.

#### Self-Regulation

Self-regulation was measured via three subscales from the behavior assessment system for children (BASC-2; Reynolds and Kampaus 2004). The BASC-2 is a widely used parent-report measure designed to measure children’s behavioral and emotional strengths and weaknesses. Past editions of this measure have not focused on children’s regulatory skill; however, the latest edition includes questions designed to measure children’s ability to regulate behavior and cognition (Reynolds and Kampaus 2004; Sullivan and Riccio 2006) and it has been used successfully in previous research as an indicator of self-regulation (e.g., McKown et al. 2009). The subscales selected for study were attentional problems, executive function, and hyperactivity. All have been shown to be valid measures for these constructs (McGlamery et al. 2007; Sesma et al. 2009; Sullivan and Riccio 2006). For example, Sullivan and Riccio (2006) compared performance on BASC scales with sub-scales from the BRIEF Parent Form and Connors’ Parent Rating Scale (CPRS), They found that these BASC scales correlated highly with other measures of self-regulation (e.g., *r* = 0.80 for BRIEF Behavioral Rating Index; *r* = 0.68 for CPRS Cognitive problems/inattention). The measure has sound psychometric properties. The median internal consistency for composites is 0.90–0.91, test–retest reliability is .84, and groups of children with preexisting clinical diagnoses tend to have distinct profiles.

Two separate, though quite similar, versions of the BASC-2 were administered to parents based on the target child’s age. Parents with children younger than 6 were administered the 24-item BASC-2: PRS-P while parents of children six and older were administered the 22-item BASC-2: PRS-C. For the current study, only items that were present on both scales were used, which resulted in a 20-item scale (α = 0.86). Parents were asked how often their child exhibited certain behaviors (e.g., how often their child interrupted conversations, paid attention, is easily distracted) on a four point Likert scale 1 (*never*) to 4 (*almost always*). Responses were summed for each parent report to create a score of children’s self-regulation, with *lower* scores indicating better self-regulation: *M* = 29.96, SD = 15.17.

### Analytic Approach

A series of regressions were conducted using STATA11. Data were weighted to approximate the US population. We used the survey weight correction in STATA to eliminate problems arising from incorrect standard error estimations (Winship and Radbill 1994). Due to deviations from a normal distribution for authoritative and authoritarian parenting, we used transformed values in analyses. To ease interpretation, the non-transformed values are displayed in the table of means (see Table 1).

## Results

A table of zero-order correlations is presented in Table 2. In the regression analyses, we were interested in isolating those demographic and parenting variables that were most strongly linked to children’s self-regulation skills. Additionally, based on previous research (Entwisle et al. 2007), an interaction term between gender and low-income was included to determine if male children from low-income families were more likely to have self-regulatory difficulties. See Table 3 for a full accounting of the regression analyses.

**Table 2:** Zero-order correlations for all variables

Variable	1	2	3	4	5	6	7	8	9	10	11
1. Self-regulation	1	−0.01	0.01	−0.03	−0.02	0.21***	−0.17**	0.03	0.20***	0.18***	−0.15**
2. *Father respondent*		1	−0.11**	−0.06	0.05	−0.04	0.11*	−0.13*	−0.13*	0.02	0.06
3. *Non*-*parent respondent*			1	0.05**	−0.03	0.11**	−0.14***	0.02	0.13**	−0.01	0
4. *Home language*				1	−0.07	−0.05	−0.07	−0.1	−0.02	0.05	0.09*
5. *Childcare*					1	0.03	0.28***	0.05	−0.21***	0.04	−0.23***
6. *Special needs*						1	−0.13**	0.02	0.11*	0.10^+^	0.06
7. Parent Education							1	−0.23***	−0.43***	−0.05	0.04
8. *Household composition*								1	0.34***	0.04	0.10^+^
9. *Family income*									1	0.07	0.01
10. *Child gender*										1	−0.04
11. Child age											1
12. *Child race: W/NL*											
13. *Child race: W/L*											
14. *Child race: AA/L*											
15. *Child race: AA/NL*											
16. *Child race: Asian*											
17. *Child race: NA*											
18. *Child race: Other*											
19. Permissive score											
20. Authoritative score											
21. Authoritarian score											

Variable	12	13	14	15	16	17	18	19	20	21
1. Self-regulation	−0.05	0.09	0	−0.04	−0.04	0	0.06	0.44***	−0.15**	0.23***
2. *Father respondent*	0.07	−0.03	0.11***	−0.10^+^	0.11^+^	0.01	−0.07	−0.06	−0.25***	0.04
3. *Non*-*parent respondent*	0.03	−0.08***	0.02	0.07^+^	−0.03	0.01	−0.03	−0.02	−0.01	−0.03
4. *Home language*	0.32***	−0.22***	0.03	0.12***	−0.31***	0.02	−0.27***	−0.05	0.02	−0.13*
5. *Childcare*	0.08	−0.05	0.02	0.13*	0	0.01	0.04	−0.03	0	−0.01
6. *Special needs*	−0.03	0.12^+^	0.03	−0.08*	−0.06	−0.02	0.04	0.21**	−0.04	0.04
7. Parent education	0.09^+^	−0.11^+^	−0.06*	−0.11**	0.19*	−0.05	0.04	−0.18	−0.06	−0.15**
8. *Household composition*	−0.26***	0.1	−0.02	0.31***	0.05	−0.01	−0.08^+^	0.09	0.11*	0.09^+^
9. *Family income*	−0.22***	0.13*	0.04	0.23***	−0.07^+^	0.06*	−0.07	0.17**	0.07	0.14**
10. *Child gender*	0.02	0.06	0.04	0.03	−0.07	−0.04	−0.13*	0.06	−0.10^+^	0
11. Child age	−0.01	−0.01	−0.08	0.04	0.04	−0.01	−0.02	−0.08^+^	−0.06	−0.01
12. *Child race: W/NL*	1	−0.51***	−0.10**	−0.48***	−0.20***	−0.14***	−0.33***	−0.16**	−0.10^+^	−0.16**
13. *Child race: W/L*		1	−0.04	−0.17**	−0.07	−0.05	−0.12*	0.09	0.04	0.06
14. *Child race: AA/L*			1	−0.04	−0.02	−0.01	−0.02	0	0.02	0.06
15. *Child race: AA/NL*				1	−0.07	−0.05	−0.11	0.09	0.14**	0.12*
16. *Child race: Asian*					1	−0.02	−0.05	−0.04	−0.03	0.06
17. *Child race: NA*						1	−0.03	0.01	0.01	0.04
18. *Child race: Other*							1	0.08	−0.06	0
19. Permissive score								1	−0.03	0.32***
20. Authoritative score									1	−0.10*
21. Authoritarian score										1

Self-regulation is coded such that *lower* scores indicate better self-regulation; Variables in italics are binary variables; *W* white, *AA* African American, *L* Latino, *NL* not Latino, *NA* Native American

^+^ *p* < .10, * *p* < .05, ** *p* < .01, *** *p* < .001

**Table 3:** Regression predicting self-regulation with demographic and parenting indicators

Variable	Step 1		Step 2		Step 3		Step 4		Step 5	
	*B (SE)*	*β*	*B* (SE)	*β*	*B* (SE)	*β*	*B* (SE)	*β*	*B* (SE)	*β*
Constant	30.17 (1.27)		34.55 (3.77)		34.34 (3.89)		26.28 (4.50)		26.27 (4.46)	
Rspdnt = father	−0.09 (.95)	0.00	0.28 (.97)	0.01	0.67 (.90)	0.03	0.07 (.79)	0.00	0.10 (.79)	0.01
Rspdnt = other	−0.62 (1.57)	−0.01	−1.89 (1.66)	−0.04	−1.28 (1.72)	−0.03	−0.32 (1.66)	−0.01	−0.12 (1.63)	0.00
Language (English = 1)	−0.70 (1.28)	−0.02	−0.83 (1.31)	−0.03	0.79 (1.57)	0.03	1.19 (1.48)	0.04	1.20 (1.47)	0.04
Special needs (yes = 1)	5.52 (1.43)	0.21***	4.74 (1.42)	0.18***	4.53 (1.41)	0.17***	2.60 (1.30)	0.10*	2.61 (1.30)	0.10*
Childcare (yes = 1)	−0.40 (.84)	−0.02	0.76 (.81)	0.04	0.02 (.8)	0.00	0.16 (.71)	0.01	0.16 (.71)	0.01
Family income (low income = 1)			3.03 (1.19)	0.16*	2.99 (1.18)	0.16*	2.38 (1.10)	0.13*	3.49 (1.49)	0.18*
Household composition (single = 1)			−1.20 (1.35)	−0.05	−0.24 (1.36)	−0.01	−0.21 (1.19)	−0.01	−0.23 (1.19)	−0.01
Parent education			−0.38 (.22)	−0.11^+^	−0.30 (.20)	−0.09	−0.14 (.19)	−0.04	−0.16 (.18)	−0.05
Child gender (1 = male)					2.51 (.82)	0.15**	2.11 (.76)	0.12**	2.68 (.86)	0.16**
Child age (months)					−0.06 (.02)	−0.16***	−0.05 (.02)	−0.13***	−0.06 (.02)	−0.14***
Child race: AA/NL					−1.14 (1.41)	−0.05	−2.03 (1.26)	−0.08	−1.95 (1.25)	−0.08
Child race: AA/L					−3.85 (5.01)	−0.04	−3.53 (4.28)	−0.04	−3.29 (4.10)	−0.03
Child race: W/L					0.76 (1.38)	0.03	0.25 (1.18)	0.01	0.25 (1.16)	0.01
Child race: Asian					1.04 (2.62)	0.02	0.46 (2.34)	−0.08	0.56 (2.34)	0.01
Child race: NA					−0.51 (1.95)	−0.01	−1.18 (1.51)	−0.02	−1.28 (1.46)	−0.02
Child race: other					2.81 (1.33)	0.09*	1.44 (1.75)	0.04	1.49 (1.76)	0.05
Authoritative parenting							−5.26 (1.92)	−0.11**	−5.20 (1.92)	−0.11**
Authoritarian parenting							1.59 (.78)	0.09*	1.48 (.79)	0.09^+^
Permissive parenting							4.49 (.73)	0.34***	4.52 (.73)	0.35***
Gender x low income									−2.06 (1.77)	−0.09
Δ*R* ^2^	0.045		0.042		0.052		0.147		0.003	

Self-regulation is coded such that *lower* scores indicate better self-regulation; *AA* African American, *W* white, *L* Latino, *NL* Not Latino, *NA* Native American; lower self-regulation scores reflect better self-regulation

^+^ *p* < .10, * *p* < 0.05, ** *p* < 0.01, *** *p* < .001

The first step in the model controlled for whether the respondent was the child’s father, another adult who was neither the father nor mother, whether English was the only language spoken in the home, whether the child had special needs, and if the child was in childcare. This first step in the analysis was significant, *F*(5, 1,136) = 3.05, *p* < 0.01, *R* ^2^ = 0.05 (see Table 3). Of the variables entered, the only one that was significantly linked with reported self-regulation was whether the child was reported to have an identified disability (β = 0.21, *p* < 0.001).

The second step of the model added the three family level demographic variables, low-income status, household composition, and parent education. With the addition of these variables, the model continued to account for a significant amount of variance, *F*(8, 1,133) = 4.54, *p* < .001, *R* ^2^ = 0.09, Δ*R* ^2^ = 0.04. Children from low-income homes had more difficulty with self-regulation (β = 0.16, *p* < .05) while children whose parents had more formal education were marginally less likely to have self-regulation problems (β = −0.11, *p* = .08). Household composition was unrelated to self-regulation.

The third step in our model introduced child-level demographic variables (i.e., child gender, child age, child race/ethnicity). The model remained significant, *F*(16, 1,125) = 3.77, *p* < 0.001, *R* ^2^ = 0.14, Δ*R* ^2^ = 0.05. Younger children (β = −0.16, *p* < .01) and male children (β = 0.15, *p* < 0.01) had weaker self-regulation skills. In addition, children from races categorized as other had lower self-regulation skills (β = 0.09, *p* < 0.05) when compared to White, non-Latino children. Adding the child-level variables reduced the association between parent’s education and self-regulation to non-significance (β = −0.09, *p* = .14).

We included variables related to parenting style in the fourth step of the model.^1^ The model with these variables included was significant, *F*(19, 1,122) = 9.00, *p* < 0.001, *R* ^2^ = 0.29, Δ*R* ^2^ = 0.15, with each of the parenting style variables accounting for a significant amount of variance in the dependent variable. The strongest of these relationships was with permissive parenting (β = 0.34, *p* < 0.001), while both authoritative parenting (β = −0.11, *p* < .01) and authoritarian parenting (β = 0.09, *p* < 0.05) were also significant. Both permissive and authoritarian styles are associated with weaker self-regulation skills whereas the reverse was found for authoritative parenting. The addition of these variables reduced the association between race and self-regulation to non-significance (β = 0.04, *p* = 0.41).

The last step included the addition of an interaction term. As suggested by Entwisle et al. (2007), we tested whether boys from low-income homes had more difficulty with self-regulation (low-income boys = 1). While the model remained significant, *F*(20, 1,121) = 8.55, *p* < 0.001, *R* ^2^ = 0.29, Δ*R* ^2^ = 0.003, the interaction term was not significant (β = −0.09, *p* = 0.25).

## Discussion

Self-regulation has emerged as a central variable to evaluate when researching processes related to how children learn and adapt to formal school settings (Blair 2002; McClelland et al. 2007). The importance of self-regulation is evidenced by the numerous research studies that have investigated correlates and predictors of self-regulation (e.g., Grolnick and Ryan 1989; Kochanska et al. 2001; Kopp 1982; Matthews et al. 2009) as well as the numerous studies that have investigated the predictive role of self-regulation across a host of outcomes (e.g., Howse et al. 2003; McClelland et al. 2007; Sektnan et al. 2010). Despite clear evidence for evaluating self-regulation in early childhood, there is surprisingly no published research utilizing a nationally representative probability sample of the American population to investigate correlates of self-regulation thus limiting generalizability to the population writ large. We sought to address this gap by utilizing data from a nationally representative survey of English-speaking American parents of a child between the ages of 2 and 8 years (*n* = 1,141) to investigate those variables frequently associated with self-regulation. In this research, we broadly defined self-regulation as a set of acquired, intentional skills involved in controlling, directing, and planning one’s cognition, emotions, and behaviors (Schunk and Zimmerman 1997). Our goal was to investigate variables frequently associated with self-regulatory behaviors in early childhood. By understanding which variables are consistently associated with regulatory abilities, interventions can more accurately target at-risk children and researchers can continue to investigate regulatory abilities with a firmer grasp on which variables are critical to measure.

The results of this study suggest that researchers should pay particular attention to child age, child gender, family income, and parenting style as each of these variables remained significant correlates of self-regulation when controlling for all other variables in the model. Notably, the addition of parenting style to the model explained the largest portion of variance with permissive parenting emerging as the most robust correlate of self-regulation. Each is discussed in more detail below.

### Demographic Variables Associated with Self-Regulation

The demographic variables studied here represented both family-level variables and child-level variables. For the family-level variables, we expected that increased parent education would be associated with stronger self-regulatory abilities while living in a low-income or single-parent household would be associated with poorer self-regulation. For the child-level demographic variables, we expected that older children would demonstrate stronger self-regulatory skills while being male or a minority would be associated with poorer self-regulation. Finally, based on suggestions that males from low income families may be particularly at risk for regulatory challenges (Entwisle et al. 2007; Morrison et al. 2009), we asked whether income status interacted with gender on self-regulation.

When controlling for all other variables in the model, family income was the only family-level demographic variable associated with self-regulation. Specifically, children from low-income backgrounds experienced greater regulatory challenges than their peers from more affluent backgrounds. As both parent education and income-to-needs ratios are frequently used as proxy measures for socioeconomic status, this finding is interesting. Our data suggests that that it is not so much the educational climate of the home but rather the economic climate of the home that is associated with children’s self-regulation. This finding lends support to Sektnan et al.’s (2010) argument that children in low-income homes have fewer resources available to promote and practice regulatory skills as well as to research which links low-income status with alterations in prefrontal cortex functioning and the cognitive processes underlying regulation (Kishiyama et al. 2009).

Our findings help clarify the literature on the role of household composition and self-regulation. Murry and Brody (1999) have suggested that children from single-parent families may struggle with self-regulation while research by Evans and Rosenbaum (2008) has suggested that household composition works through other variables. Our work found no relationship between household composition and self-regulation. If household composition does play a role, its association was captured through other variables in our models.

The results for our child-level demographic variables also yielded interesting results. As expected, children’s age was significantly correlated with self-regulation. Children’s self-regulatory skills become more sophisticated as they get older. This finding remains when controlling for all other variables in the model, and is consistent with previous research on age and self-regulation (Kopp 1982; Raffaelli et al. 2005). Findings for children’s gender also supported our hypothesis. When controlling for all other variables, girls were found to have stronger self-regulation skills than boys. The interaction between gender and low income status was not significant, suggesting that the relationship between gender and self-regulation is not differentially impacted by income status.

Although our hypotheses for child’s age and gender were supported, our findings for child’s race/ethnicity were inconsistent with predictions. Previous research on race and ethnicity suggests that minority status may be a risk factor for lower self-regulation (Sektnan et al. 2010). We found conflicting results in our analyses. Children who were identified as White-Latino, Black-Latino, Black-non Latino, Asian, Native American, and other races performed similarly to the White-non Latino reference group. Furthermore, coefficients suggested that African Americans (both Latino and non-Latino) and Native Americans actually held higher self-regulation skills compared to the White-non Latino reference group. Because the survey was administered in English, it is unsurprising that there were no particular trends for Latino status. However, our findings related to race contradict previous research. A review of the correlations across independent variables helps explain this finding. When looking at the correlation matrix (see Table 2), we see that African American children were significantly more likely to live in a low-income home when compared with White-non Latino children. Recall that low income children had more difficulty with self-regulation. This relationship suggests that it is not race per se that correlates with self-regulation but rather it is the contextual influence of family income that matters.

### Parenting Variables Associated with Self-Regulation

Previous research examining parenting styles suggested that certain types of parenting play a role in children’s self-regulation (Crossley and Buckner 2011; Karreman et al. 2006). Based on this and other research, we expected authoritative parenting to be associated with improved regulatory skills while authoritarian parenting would be associated with lower regulatory skills. These hypotheses were confirmed. In previous meta-analytic research, authoritarian parenting was found to be a stronger predictor of self-regulation than authoritative parenting. Here, we found authoritarian parenting to be a less robust correlate of self-regulation. This may be due to the somewhat low internal consistency for the authoritarian parenting scale. Low internal consistency reduces our power to detect effects, thus any relationship between the authoritarian scale and self-regulation is likely underestimated (Sutcliffe 1958).

Based on work by Bernier et al. (2010), we hypothesized that a permissive parenting style would be associated with decreased regulatory skills in young children. This hypothesis was at odds with the one study looking at permissive parenting and self-regulation (Morris 2003); however, it mapped onto other research investigating relationships among permissive parenting and child outcomes. Not only was permissive parenting linked to greater struggles with self-regulation thus supporting our hypothesis, it was *the* most powerful variable in the model (β = 0.34). To put this in perspective, the next strongest relationship was with child age (β = 0.13).

Our findings for parenting style support work by Bernier et al. (2010). Parents who rely on nurturing parenting practices that reinforce the child’s sense of autonomy, while still maintaining a consistent parenting presence, have children who demonstrate stronger self-regulatory skill. Parents who exert an excess of parental control (i.e., authoritarian parents) have children with weaker self-regulatory skill. And finally, our results suggest that parents who have notable absence of parental control (i.e., permissive parents) are more likely to have children with considerable regulatory deficits.

### Study Limitations

Our reliance on cross-sectional data comes with caveats that we cannot address causality nor infer direction of relationships. Additionally, we were limited to households where at least one adult was a fluent English speaker. Thus, approximately 2 % of all homes in our targeted sample were ineligible to participate (S. Sherr, personal communication, January 21, 2009). The decision to exclude non-English speaking homes was driven by the prohibitive costs associated with translating the measures and training interviewers to conduct the survey in other languages. Future research should make an effort to illuminate how children raised in non-English households are similar/different from English speaking homes.

There was no second person verification in this study. Data were based on the response of one parent. While some large sample studies (e.g., Child Development Supplement for the Panel Study of Income Dynamics) have worked to incorporate multiple voices as a means of triangulation and verification, such incorporation is quite costly and was not a feasible option for a telephone survey. We did attempt to alleviate this concern by ensuring that the interviewee was the individual who spent “most of the time directly caring for the child” and thus would be best suited to answering questions about the child. Additionally, we relied on parental report of children’s self-regulation as opposed to using a measure in which we work directly with the child (e.g., delay of gratification). By choosing to conduct a large scale representative survey, we had to acknowledge that there would be some measurement loss. However, we believe that what we lost in measurement was gained in generalizability.

## Conclusion

In this research, we investigated how demographic and parenting variables relate to self-regulation in early childhood. The variables included in the model are by no means an exhaustive account of the potential correlates of self-regulation; however, they do represent many of the most common variables included in discussions of self-regulation predictors. Our results replicate and extend previous research. This replication is an important contribution as it bolsters the claims made by previous research using a generalizable data set. Moreover, our unexpected findings for minority status and parent education offer important contributions for future research studies.

Perhaps the most immediate and important take-away relates to how our study can inform policy related to children’s regulatory development. As other scholars have noted (Lohr 2010; Mutz 2011), if researchers wish to make policy suggestions or recommendations for the population at large, it is vitally important that the sample used to study a particular phenomenon accurately map onto the larger population. While convenience and other non-representative samples frequently illuminate the relationships between targeted variables (perhaps even more efficiently than a large representative survey can, Chang and Krosnick 2009), there are significant issues regarding whether we should assume that the ‘average’ child and family behaves the same way. The methodology employed in our study can support the policy recommendations that researchers and practitioners make. For example, because we are able to make claims regarding our sample’s ‘representativeness’ to the larger American populace, we have greater confidence that when policy makers or child/family advocates design interventions to support the development of children’s self-regulation skills, they are targeting those populations most in need of support (e.g., children from low income families, boys).

With this in mind, another important implication from this study is that parenting style appears to be an important correlate of children’s regulatory abilities. Of the variables in our model, permissive parenting had *the* strongest association with self-regulation skills in children. The more permissive a parent was (in terms of hesitancy to follow-through with the child) the less self-regulation was exhibited by the child. More research using causal models is necessary to better understand this relationship, as is research on how parenting styles in multi-parent households may interact to support or suppress children’s regulatory behaviors (Volling et al. 2006). For now, this research suggests that educating parents about the benefits of ‘following-through’ and maintaining appropriate limits with their child would offer an important contribution to the healthy development of young children. And although we did not assess the role secondary or tertiary caregivers play in shaping regulation, this advice may have import for professionals who work directly with children and/or their caregivers in child-care settings.

## Footnotes

1 Baumrind’s (1971) research conceptualized parenting styles categorically whereas this measurement approach adopts a dimensional approach such that each parent is assigned a score on each of the three styles. We conducted additional analyses to ensure that our dimensionally-based findings would be similar to a categorical approach. Following procedures described by Stephenson et al. (2010), we categorized individuals as purely “authoritarian”, “authoritative”, or “permissive”. To do this, individuals with scores above the median on authoritarian parenting and below the median on permissive parenting and authoritative parenting were categorized as authoritarian. The same procedure was replicated for permissive and authoritative. This resulted in 148 individuals categorized as purely authoritarian, 92 categorized as purely permissive, and 248 individuals categorized as purely authoritative. Individuals who did not fall into one of these categories (*n* = 653) were removed. Regression analyses were conducted with the categorical variable as opposed to the three continuous variables. Results were replicated. Children with permissive parents and authoritarian parents were significantly more likely than children with authoritative parents to have regulatory challenges.
